# Meteorology as Study and Pastime

**Published:** 1907-12-28

**Authors:** 


### The Practitioner's Relaxations and Hobbies.

### METEOROLOGY AS STUDY AND PASTIME.

Among many interesting pursuits, the study of meteorology stands high in the list as one that will form a most useful relaxation for a medical man, and one peculiarly suited to him, not only on account of its importance in relation to general health, but also because it requires the exercise of certain qualities which, as a rule, form distinctive features in a doctor's character—namely, order, exactness, and patience. To gain proficiency in any science it is necessary to be earnest and thorough in its pursuit; and the study of the weather in like manner must be approached with full determination to carry out faithfully all its requirements. It is not a work that may be taken up one day and put down the next. Once undertaken, it exacts absolute reliability, for without this no good results can be obtained. In taking observations, for instance, great punctuality is necessary at the hours fixed, and no deviation is permissible. The readings must be made as accurately as possible, and blow rain or snow, in mist, fog, or darkness, no shirking must be evinced; every duty must be gone through as carefully as at the most favourable time. The records obtained must be entered neatly and fully, and it is always best to do this at once, as any error found in the note-book can often be rectified from memory, or, if necessary, by returning to make a fresh observation. But in spite of the apparently exacting nature of the work, one quickly develops a love for it, and the more fully one enters into it, the stronger is its attraction.

It may pertinently be asked by anyone not yet initiated in the work, "What purpose does it serve?" Of course, the chief purpose of meteorology as a science is to give us information respecting climate generally, and by climate we mean the conditions of any place in relation to its various atmospheric phenomena, such as temperature, wind, rainfall, etc. But in addition to this general knowledge we want that of "individual" climates, we want to learn about the climate of the place in which we find ourselves located. This can only be obtained by prolonged and systematic observations on the spot and by comparison of such statistical facts as can be thus collected. Such a study is important, and cannot be over-estimated, considering how marked is the influence of weather upon human health. In addition, however, to its utility in furnishing us with the necessary information, its study will serve another useful purpose; it will afford just that relaxation of mind in the change of work that a medical man requires, and the interest aroused in observing Nature and in endeavouring to wrest her secrets from her will be found most invigorating. But, further still, the study of meteorology holds out great inducements for those interested in research as it presents various problems which have not yet been satisfactorily solved, and certain branches, too, of the subject have received less attention than others from the bulk of observers. Here, therefore, one may find an open field of work, and by painstaking and successful investigation, help to promote an advancement in the science.

There is a peculiar fascination in noting the various aspects of the weather. The sky, in its ever-changing character, flecked by clouds, with their rapidly alternating tints and colours, and their multiple forms and movement, presents a marvellous picture to the student of nature. Various natural phenomena may be noted, giving indications that enable us to make a forecast, more or less correct.

There is already a vast network of observing stations all over the British Isles, under the supervision of the Royal Meteorological Society, in charge of a band of voluntary observers, but there is still room for more. Owing to irregularity of distribution numerous places do not come within the range of these stations, and there is therefore plenty of scope for any one to take up this attractive pastime. Wherever a doctor is settled it is of importance to him to know the climate of that place, particularly with respect to the amount of rainfall, hours of sunshine, temperature, and mean direction of wind, and by personal observation to obtain statistical records of the same. As regards taking observations, of course, each one will follow his own choice. If it be desired to make them solely for private purposes, as a matter of personal interest and amusement, a very good plan is to construct charts and diagrams: one can more easily determine the actual atmospheric conditions by a study of these than by looking through long columns of figures in a monthly record. Or, if we desire to go a step further, with a view to help in general public work, we can form what is known as a second-order or climatological station, where observations must be taken twice daily, at 9 A.M. and 9 P.M., of air-pressure, temperature—the daily maxima and minima, dry and wet bulb readings—wind, cloud, weather, rainfall, with general daily remarks on the weather. All instruments used must be good and accurate in their working, and must have been verified at Kew. If approved by the Society, and all notes are carefully tabulated and sent every month to the secretary, they will appear in the quarterly official publication. Of course, in the former method, it is not necessary to have so many instruments, and one observation daily at 9 A.M. will suffice. Some initial expense will be incurred, as one must be provided with suitable instruments to make accurate observations. It is best, therefore, to limit oneself, if expense is a consideration, to such as are absolutely necessary, and to have the best. A very useful little work, "Hints to Observers," by Mr. W. Marriott, price 1s. 6d., gives full directions as to "how to observe," the instruments needed, and how to arrange them. With this capital aid one can undertake the work without difficulty. If any are led by these remarks to take up the study they will find themselves amply repaid by the new interest it will create, and by the pleasure, as well as the distraction from the more strenuous labours of the day, that it will afford.

